# Genome-Wide Analysis of Chemosensory Protein Genes (CSPs) Family in Fig Wasps (Hymenoptera, Chalcidoidea)

**DOI:** 10.3390/genes11101149

**Published:** 2020-09-29

**Authors:** Zhaozhe Xin, Dawei Huang, Dan Zhao, Jiaxing Li, Xianqin Wei, Jinhua Xiao

**Affiliations:** Institute of Entomology, College of Life Sciences, Nankai University, Tianjin 300071, China; 1120180392@mail.nankai.edu.cn (Z.X.); huangdw@nankai.edu.cn (D.H.); 2120181038@mail.nankai.edu.cn (D.Z.); 2120181034@mail.nankai.edu.cn (J.L.); weixq@nankai.edu.cn (X.W.)

**Keywords:** chemosensory proteins, fig wasps, whole-genome level

## Abstract

Chemosensory proteins (CSP) are a class of acidic soluble proteins which have various functions in chemoreception, resistance and immunity, but we still have very little knowledge on this gene family in fig wasps, a peculiar insects group (Hymenoptera, Chalcidoidea) that shelter in the fig syconia of *Ficus* trees. Here, we made the first comprehensive analysis of CSP gene family in the 11 fig wasps at whole-genome level. We manually annotated 104 CSP genes in the genomes of the 11 fig wasps, comprehensively analyzed them in gene characteristics, conserved cysteine patterns, motif orders, phylogeny, genome distribution, gene tandem duplication, and expansion and contraction patterns of the gene family. We also approximately predicted the gene expression by codon adaptation index analysis. Our study shows that the CSP gene family is conserved in the 11 fig wasps; the CSP gene numbers in pollinating fig wasps are less than in non-pollinating fig wasps, which may be due to their longer history of adaptation to fig syconia; the expansion of CSP gene in two non-pollinating fig wasps, *Philotrypesis tridentata* and *Sycophaga agraensis*, may be a species-specific phenomenon. These results provide us with useful information for understanding the evolution of the CSP gene family of insects in diverse living environments.

## 1. Introduction

Insects can recognize various chemicals’ cues from the external environment, through their sensitive chemical receptors, to complete the process of foraging, spawning, mating, avoiding predators and finding hosts [1,2,3]. Chemical perception of insects is divided into two major processes: gustation and olfaction. The gustation recognizes soluble chemicals such as sweet, bitter substances and amino acids, which induces feeding behaviors [4]. The olfaction that can be used to find food, predators and mates usually recognizes volatile chemical cues [5]. Several kinds of proteins have been confirmed to be involved in chemosensory perception, including gustatory receoptors (Gr), olfactory receptors (Or), ionotropic receptors (Ir), sensory neuron membrane proteins (SNMPs), odorant degrading enzyme (ODE), odorant binding proteins (OBPs) and chemosensory proteins (CSPs) [6,7], in which OBPs and CSPs participate in the first step of chemical molecules recognition [8,9]. External hydrophobic chemical molecules first enter the sensillum lymph through the epidermal pores, and are then captured by water-soluble OBPs/CSPs and transported to member-bound Grs, Ors and IRs [10,11]. OBPs and CSPs are two similar types of carrier proteins in dissolving and transmitting chemical signals or lipophilic compounds to chemosensory receptors [12,13,14], with the difference that OBPs react with volatile small odorant molecules, while CSPs are acid soluble proteins that combine and transport liposoluble compounds [15,16,17].

The first discovered CSP gene was in the antenna of *Drosophila melanogaster*, and it was named olfactory specific protein D (OS-D) and pheromone-binding proteins A-10 (A-10), due to its preferred expression in the antennae [18,19]. CSPs are a kind of water-soluble protein widely expressed in insects with lower isoelectric point (pI) and molecular weight (MW) [20,21]. CSPs possess four conserved cysteines and conserved cysteine pattern (C1-X_6-8_-C2-X_16-21_-C3-X_2_-C4), with adjacent cysteines forming two disulfide bonds, creating a stable structure with two small rings (C1-X_6-8_-C2, C3-X_2_-C4) [22,23]. The tertiary structure of CSPs consists of six α-helices, forming a hydrophobic pocket-like structure for binding ligands. The CSP gene family is more conserved across insects, evolutionary less dynamic and with fewer members than the OBP family [24].

Xiao et al. [25] have sequenced, assembled and analyzed the genome and transcriptome of the pollinating fig wasp species of *Ceratosolen solmsi* (Hymenoptera: Chalcidoidea) and have discovered seven CSP genes in the genome of this species. However, the CSP gene family has not been thoroughly analyzed at the genome-wide level in other fig wasp species, especially when the divergent life histories of pollinating and non-pollinating fig wasp species are considered. Both pollinating and non-pollinating fig wasp species are associated with figs, which have to fulfil their developmental processes within the compact fig syconia; upon maturation, pollinating fig wasps finish their mating process mainly within the same fig syconia where they have grown, while non-pollinating fig wasps will fly out to mate [26]. Considering that the environment inside the fig syconia is simpler than the environment outside the fig syconia, this difference in life history indicates a simpler environment than the pollinating fig wasps have experienced, suggesting that pollinating fig wasps may have a reduced gene family of CSPs. We have obtained the genomes and transcriptomes data of the 11 fig wasps, including six pollinating fig wasp species, namely *Eupristina koningsbergeri*, *Platyscapa corneri*, *Kradibia gibbosae*, *Ceratosolen fusciceps*, *Dolichoris vasculosae* and *Wiebesia pumilae*, and five non-pollinating fig wasp speices, namely *Apocrypta bakeri*, *Philotrypesis tridentata*, *Sycobia* sp.2, *Sycophila* sp.2 and *Sycophaga agraensis*. These data make it possible for us to study CSP gene family of fig wasps. In this study, we wanted to investigate the composition and characteristics of CSP gene family in the genomes of the 11 fig wasps. Furthermore, the differences in CSP gene family between pollinating and non-pollinating fig wasps were further explored. This work presents a comprehensive analysis of the CSP gene family in the 11 fig wasps and provides useful information for the study of chemosensory systems of fig wasps.

## 2. Materials and Methods

### 2.1. Genome Sequence Sources

The analysis was mainly based on the genomes and transcriptomes of the 11 fig wasps. The genome sequences data were deposited into the NCBI database, with project accession IDs of PRJNA641212 and PRJNA494992. In addition, seven CSP genes of the species of *C. solmsi* were obtained from its published genome [25]. Nine CSP genes of *Nasonia vitripennis* were obtained from the official gene set [27]. Six CSP genes of *Apis mellifera* and four CSPs genes of *D. melanogaster* were obtained from published articles [24,28].

### 2.2. Manual Annotation and Identification of CSPs

To find all putative CSPs in the 11 fig wasps, we performed manual annotation. The CSP genes of *C. solmsi*, *N. vitripennis*, *A. mellifera* and *D. melanogaster* were regarded as the seed sequences. The tblastn search was performed for candidate CSP genes with significant hits (*E*-value < 10^−5^). The e-value was raised when protein sequences were short and few blast hits were found. The positions of each exon were verified and corrected according to the actual transcription shown in the transcriptome, using the IGV software. Nucleotide sequences were obtained, using the BioEdit, and were translated into protein sequences, using the ExPASy website (https://web.expasy.org/translate/). The scaffold where the gene predicted to be located by tblastn and the seed protein sequences were uploaded to the softberry website (http://www.softberry.com/berry.phtml?topic=fgenes_plus&group=programs&subgroup=gfs), for exons prediction, when the gene predicted by tblastn was not transcriptionally expressed in the IGV software. We confirmed all candidate CSP genes domains against CSP conserved domain information (OS-D) with Pfam protein families database (http://pfam.xfam.org/) and Conserved Domain Database (CDD) (https://www.ncbi.nlm.nih.gov/Structure/cdd/wrpsb.cgi).

### 2.3. Phylogenetic Analyses

A phylogenetic tree was constructed with all CSP genes from the 11 fig wasp species and *D. melanogaster* to classify CSP genes. Another phylogenetic tree was constructed with the CSP genes from only the 11 fig wasp species to conduct comprehensive analyses of CSP genes, motifs, domains and gene structures. Phylogenetic analyses were based on the amino acid dataset, with sequences aligned by using MAFFT, with the default settings [29], and trees constructed by Maximum Likelihood (ML) method, using the IQ-TREE [30]. The LG+I+G model was chosen as the best model according to the results of ProtTest v3.2.1 [31], and ML analyses were performed with 1000 bootstrap replications. The resulting phylogenetic trees were visualized in FigTree v1.4.0 [32]. The bootstrap values ≥ 70% were marked on the ML trees. We used the Interactive Tree of Life (http://itol.embl.de/) to polish the phylogenetic trees [33].

### 2.4. Analysis of CSPs Characteristics

Geneious Prime v2020.0.4 was used to visualize and manually adjust the results of the multiple amino acid sequences alignment. The relative frequency of the corresponding amino acid at each position was generated, using WebLogo online website (http://weblogo.threeplusone.com/). The MW and pI of CSP genes were calculated by using ExPASy ProtParam (https://web.expasy.org/compute_pi/). Motif analysis of the CSP genes was conducted by using MEME online server (http://meme-suite.org/tools/meme) [34], with parameters set as “minimum width = 6, maximum width = 50, number of motif to find = 10”. The comprehensive analyses on the CSPs’ gene trees, motifs, domains and gene structures, as well as the distribution and tandem analyses, were all conducted by using TBtools v0.66831 [35].

### 2.5. CSP Gene Family Expansion and Contraction

CAFE v4.2.1 was used to infer the expansion and contraction of CSP gene family based on lambda values (the probability of gene gain and loss per unit of time during species evolution) [36] with default parameters. The divergence time tree was based on submitted but still unpublished data from our lab. On the basis of the 11 fig wasps, *C. solmsi*, *N. vitripennis*, *A. mellifera*, *D. melanogaster*, *Acyrthosiphom pisum* and *Daphnia pulex* were added in the divergence time tree.

### 2.6. Gene Expression Pattern Predicted by Codon Adaptation Index Analysis

To approximately predict the level of expression of these CSP genes, we estimated the codon adaptation index (CAI) values with CAIcal server (http://genomes.urv.es/CAIcal/) [37]. The codon usage table of *N*. *vitripennis*, which is closely related to the 11 fig wasps, was used as a reference species codon usage table.

## 3. Results

### 3.1. Identification of CSPs Genes in the 11 Fig Wasp Species

We identified 104 CSP genes in the genomes of the 11 fig wasp species through manual annotation. There were eight CSP genes in each of the five pollinating fig wasp species of *P*. *corneri* (PcorCSP1-8), *K*. *gibbosae* (KgibCSP1-8), *C*. *fusciceps* (CfusCSP1-8), *D*. *vasculosae* (DvasCSP1-8) and *W*. *pumilae* (WpumCSP1-8); and nine CSP genes in the pollinating fig wasp species of *E*. *koningsbergeri* (EkonCSP1-9) and in the non-pollinating fig wasp species of *Sycobia* sp.2 (SbspCSP1-9). For the other non-pollinating fig wasp species, 10 CSP genes were annotated in *A*. *bakeri* (AbakCSP1-10) and *Sycophila* sp.2 (SpspCSP1-10), and 13 CSP genes in *P*. *tridentata* (PtriCSP1-13) and *S*. *agraensis* (SagrCSP1-13) (Table 1). Among these 104 CSP genes, 101 genes had complete CSP family domain (OS-D), and the remaining three CSP genes (CfusCSP3, WpumCSP2 and SagrCSP3) had incomplete CSP family domain (OS-D) with incomplete N-terminal. We considered all 104 CSP genes as members of the CSP gene family.

### 3.2. Characterization of the CSPs Genes

The CSP gene characteristics, including scaffold localization, positions of gene start and end, gene direction, the number of exons, length of coding sequence (CDS), length of amino acid sequence, total G+C content (%G+C), the G+C content of the first codon (%G+C(1)), the second codon (%G+C(2)) and the third codon (%G+C(3)), MW and pI, were analyzed (Supplementary Materials Table S1). Among the 104 CSP genes, the number of amino acids ranged from 99 (AbakCSP1) to 237 (CfusCSP7). The levels of G+C content ranged from 31.0% (SagrCSP9) to 71.2% (SbspCSP6). The levels of G+C content of the first codon ranged from 37.3% (PtriCSP12) to 70.1% (SbspCSP6). The levels of G+C content of the second codon ranged from 26.1% (EkonCSP9) to 57.6% (KgibCSP5). The levels of G+C content of the third codon ranged from 21.5% (KgibCSP3) to 95.2% (SagrCSP1). The MWs ranged from 11.0 kDa (AbakCSP1) to 27.2 kDa (CfusCSP7). The pIs ranged from 4.4 (SbspCSP6) to 10.3 (KgibCSP1). AbakCSP1 had the smallest number of amino acids and the lowest MW. CfusCSP7 had the largest number of amino acids and the highest MW. SbspCSP6 had the highest G+C content, the highest G+C content of the first codon and the lowest pI.

### 3.3. Multiple Sequence Alignment, Phylogenetic Analysis and Classification of the CSP Genes

The result of the multiple sequences alignment is shown in Supplementary Materials Figure S1. Four conserved cysteine residues were present in the expected positions. WebLogo online website was further used to generate the relative frequency of multiple amino acids at each position. The first conserved cysteine was at position 223, the second at 232, the third at 252 and the fourth at 255. The four-cysteine patterns (C1-X_6/8_-C2-X_18-19_-C3-X_2_-C4) in the 11 fig wasps were similar to the CSP gene patterns identified in other insects [38]. Adjacent cysteines among the four cysteine residues were connected to form two disulfide bonds (C1-X_6/8_-C2 and C3-X_2_-C4). The highly conserved sequences indicate that the CSP genes may play conserved roles in fig wasps.

**Figure 1:**
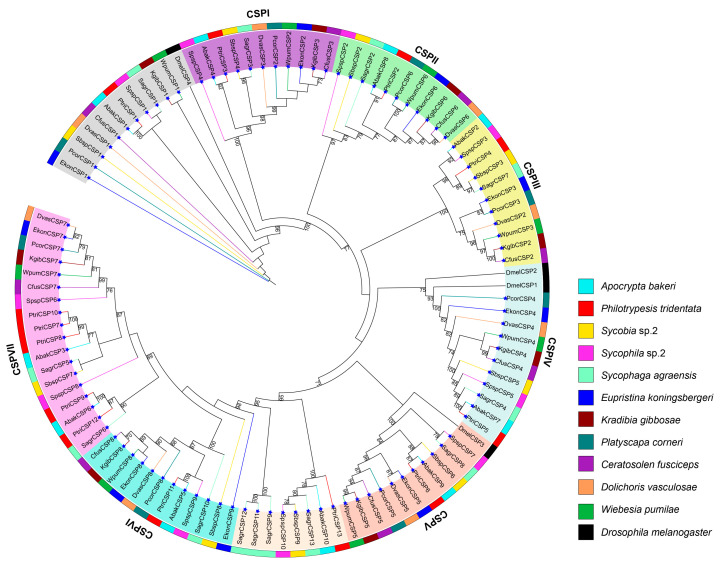
Phylogenetic tree of CSP genes from the 11 fig wasps and *D. melanogaster*. The phylogenetic tree was constructed by using the Maximum Likelihood (ML) method with 1000 bootstrap replications. Bootstrap values ≥ 70% are shown at the nodes. The CSP genes of *D. melanogaster* are abbreviated to DmelCSPs. The outermost colored rings represent the CSP genes of different species. The seven CSP groups are distinguished by different background colors. The blue star signs which are at the end of node branch represent the 104 CSP genes of the 11 fig wasps.

Phylogenetic analyses were based on amino acids of 104 CSP genes of the 11 fig wasps and four CSP genes of *D. melanogaster* using ML method. CSP genes were classified into seven mononphylic groups formed on the phylogenetic tree, including CSPI, CSPII, CSPIII, CSPIV, CSPV, CSPVI and CSPVII (Figure 1). In CSPI, CSPII, CSPIII, CSPIV and CSPV, each of the 11 fig wasps had one CSP gene. The DmelCSP3 was locateded in the CSPV group, and DmelCSP1 and DmelCSP2 were in the CSPIV group. In the CSPVI group, except for the pollinating fig wasp species of *E. koningsbergeri*, which had two CSP genes, all other fig wasps had only one CSP gene. In the CSPVII group, each of the six pollinating fig wasps and one non-pollinating fig wasp (*Sycobia* sp.2) had one CSP gene, and each of three non-pollinating fig wasps (*A*. *bakeri*, *Sycophila* sp.2 and *S*. *agraensis*) had two CSP genes, and the other non-pollinating fig wasp species of *P. tridentata* had five CSP genes.

We also compared the gene numbers between pollinating and non-pollinating fig wasps. For the six pollinating fig wasps, five species had eight CSP genes each, and *E*. *koningsbergeri* had nine. For the five non-pollinating fig wasp species, the *P. tridentata* and *S. agraensis* had 13 CSP genes, respectively, each of *A. bakeri* and *Sycophila* sp.2 had 10 CSP genes, and *Sycobia* sp.2 had nine CSP genes (Table 1). The number of CSP genes of pollinating fig wasps was significantly less than non-pollinating fig wasps (*p*-value < 0.01).

### 3.4. Comprehensive Analyses of the Gene Tree, Conserved Motifs, Domains and Gene Structures of the CSP Genes

**Figure 2:**
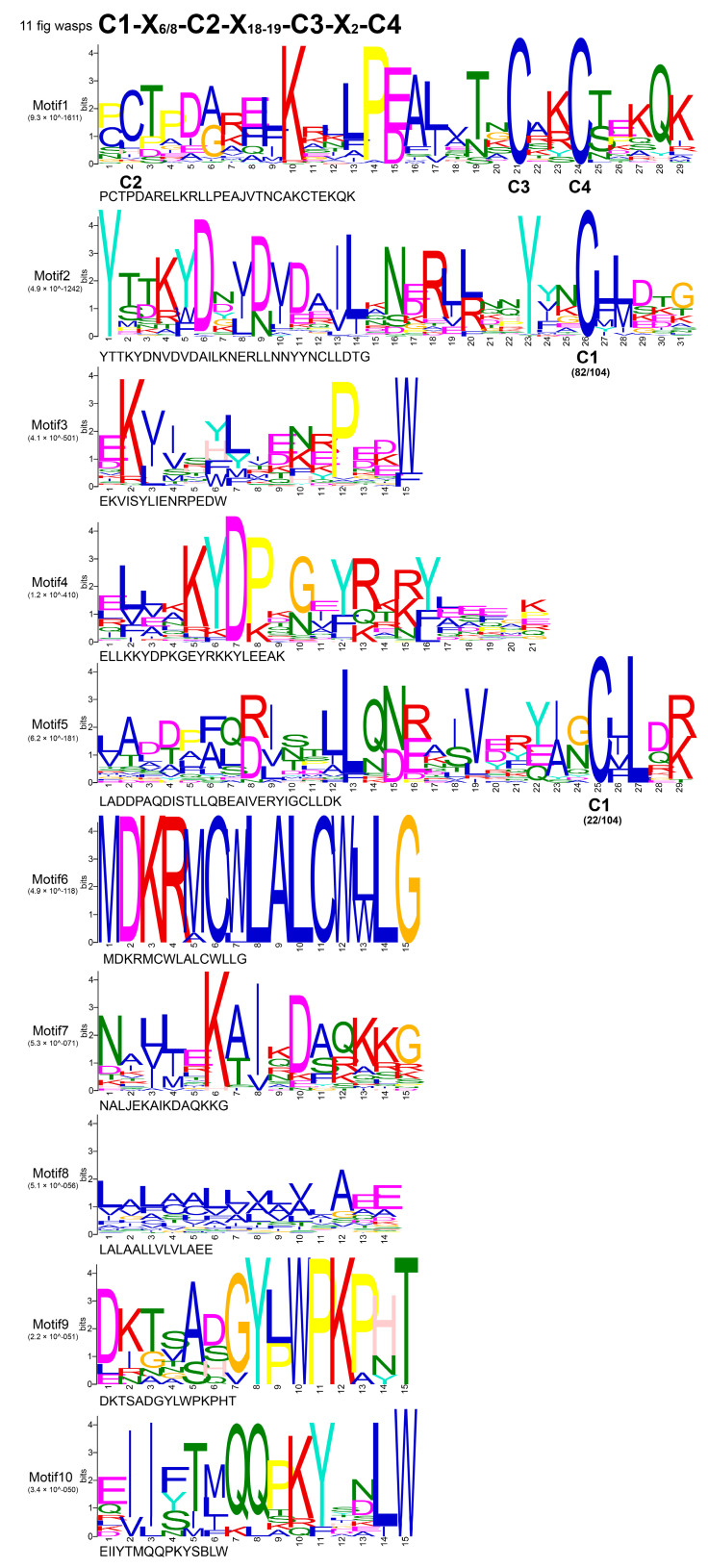
The 10 conserved motifs of 104 CSP genes in the 11 fig wasps. Four conserved cysteine residues (C1–C4) correspond to the corresponding positions of motifs. X_6/8_ indicate six or eight non-cysteine amino acids; X_18-19_ indicate 18 to 19 non-cysteine amino acids; X_2_ indicate two non-cysteine amino acids.

**Figure 3:**
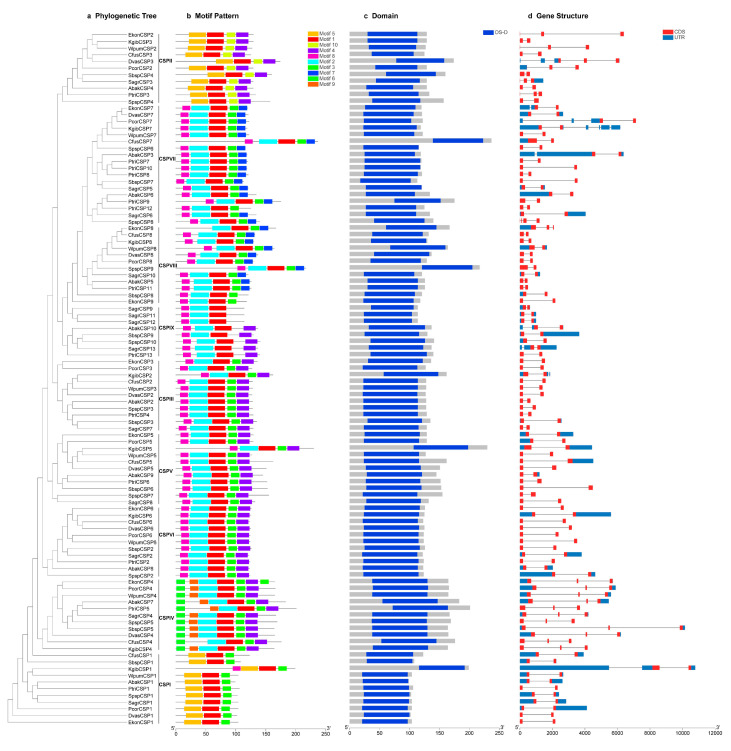
Phylogenetic relationships, conserved motifs, domains and gene structures analysis of CSP genes from the 11 fig wasps. (**a**) The phylogenetic tree is constructed based on amino acids sequences of the 11 fig wasps CSP genes using the IQ-TREE with 1000 bootstrap replications. (**b**) Distributions of conserved motifs in CSP genes. The motifs, numbers 1–10, are displayed in different colored boxes. The sequence information for each motif is provided in Figure 2. (**c**) The domains of 104 CSP genes of the 11 fig wasps. The blue boxes indicate the location of CSP family domain (OS-D) in the protein sequences; gray boxes represent the sequence regions of the non-conserved domain. (**d**) Exon–intron structures of all CSP genes from the 11 fig wasps. Blue boxes represent untranslated 5′-and 3′-regions, red boxes represent exons, and black lines indicate introns.

Details of 10 putative motifs are outlined in Figure 2 and Figure 3b. The lengths of these conserved motifs varied from 14 to 31 amino acids. Among the 104 sequences, the first of the four conserved cysteine residues appeared in motif 5 in 21.2% of the sequences, and appeared in motif 2 in the remaining 78.8% sequences. The second, third and fourth conserved cysteine residues all appeared in motif 1 (Figure 2). The conservative pattern of CSP genes in motifs was consistent with the results of the multiple sequences alignment. All the 104 CSP genes had the conserved CSP family domain (OS-D), and the location of each domain was consistent with the motif distribution of each CSP protein (Figure 3c). The number of exons varied from two to four in 104 CSP genes of the 11 fig wasps (Figure 3d). Only SpspCSP8 had four exons, 18 CSP genes (17.3%) had three exons and 85 CSP genes (81.7%) had two exons.

We mapped the motifs, domains and gene structures to the gene tree of CSPs of the 11 fig wasps for comprehensive analyses (Figure 3). All the 11 CSP genes in group of CSPI shared the same motifs order (5-1-10-4), in which six CSP genes had three exons, and five had two exons. The genes in CSPVI and CSPVII groups of closely phylogenetic relationships had similar motif patterns and numbers of exons. For example, in group of CSPVII, 17 of the 18 CSP genes shared the same motifs order (8-2-1-3-7), except that the motif order of the gene of PtriCSP12 was 8-2-1-3; 17 CSP genes had two exons, and the gene of SpspCSP8 had four exons. In the group of CSPVI, nine of the 12 CSP genes shared the same motifs order (8-2-1-3-7), whereas the motif order of EkonCSP8 was 2-1-3-7, and motif orders of SbspCSP8 and EkonCSP9 were 8-2-1-3; 11 CSP genes had two exons, and EkonCSP8 had three exons. The genes in CSPIII, CSPV and CSPII groups of closely phylogenetic relationships also had quite the same motif order (8-2-1-3-4) and the same number of exons (two exons). In CSPIV, nine of the 11 CSP genes shared the same motifs orders (6-9-2-1-3-4), and motif orders of EkonCSP4 and CfusCSP4 was 6-9-2-1-3-4-3 and 6-2-1-3-4, respectively; all the 11 CSP genes had three exons.

### 3.5. Distribution and Tandem Analysis of CSP Genes on the Genomic Scaffolds

**Figure 4:**
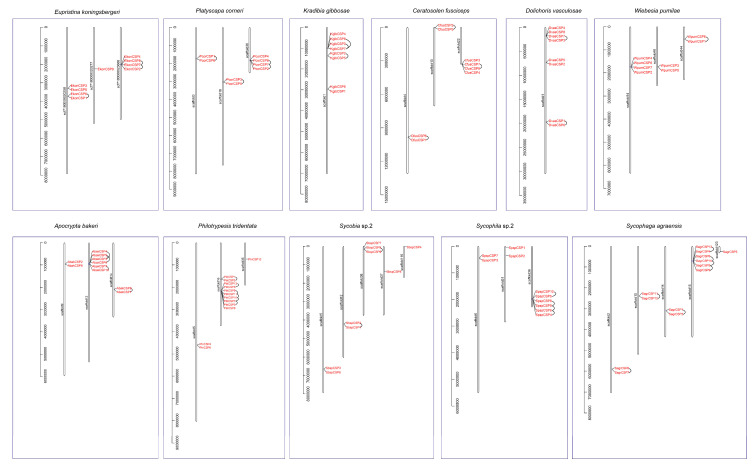
Scaffold location and gene tandem duplication of all CSP genes. The tandem duplicated genes are marked by black lines.

The distribution and tandem analysis of 104 CSP genes on the corresponding genomes’ scaffolds were further analyzed (Figure 4). In *E. koningsbergeri*, EkonCSP6 and EkonCSP1 were a pair of tandem repeat genes, distributing in scf7180000020258, and EkonCSP8, 7 and 2 were a group of three tandem duplicated genes, distributing in scf7180000023666. In *P. corneri*, two pairs of tandem repeat genes (PcorCSP1 and 6; and PcorCSP3 and 5) were distributed in scaffold3 and 18, respectively, and PcorCSP8, 7 and 2 were a group of three tandem duplicated genes, distributing in scaffold36. In *K. gibbosae*, a pair of tandem repeat genes (KgibCSP2 and 5) and a group of three tandem duplicated genes (KgibCSP8, 3 and 7) were distributed in scaffold1. In *C. fusciceps*, three pairs of tandem repeat genes (CfusCSP6 and 1; CfusCSP2 and 5; and CfusCSP7 and 8) were distributed in scaffold4, 10 and 22, respectively. In *D. vasculosae*, two pairs of tandem repeat genes (DvasCSP7 and 3; and DvasCSP1 and 6) were distributed in scaffold1. In *W. pumilae*, a pair of tandem repeat genes (WpumCSP6 and 1) were distributed in scaffold44. In *A. bakeri*, a pair of tandem repeat genes (AbakCSP7 and 10) and a group of four tandem duplicated genes (AbakCSP4, 5, 3 and 6) were distributed in scaffold12, and AbakCSP8 and AbakCSP1 were a pair of tandem repeat genes, distributing in scaffold14. In *P. tridentata*, two pairs of tandem repeat genes (PtriCSP1 and 2; and PtriCSP13 and 5) and a group of five tandem duplicated genes (PtriCSP9, 11, 10, 7 and 3) were distributed in scaffold16. In *Sycobia* sp.2, two pairs of tandem repeat genes (SbspCSP2 and 1; and SbspCSP5 and 9) were distributed in scaffold12 and 36, respectively. In *Sycophila* sp.2, a pair of tandem repeat genes (SpspCSP10 and 5) and a group of four tandem duplicated genes (SpspCSP8, 9, 6 and 4) were distributed in scaffold36. In *S. agraensis*, three pairs of tandem repeat genes (SagrCSP8 and 7; SagrCSP11 and 12; and SagrCSP1 and 2) were distributed in scaffold2, 10 and 14, respectively, and a pair of tandem repeat genes (SagrCSP13 and 4) and a group of four tandem duplicated genes (SagrCSP6, 10, 5 and 9) were distributed in scaffold15.

### 3.6. CSPs Gene Family Expansion and Contraction

**Figure 5:**
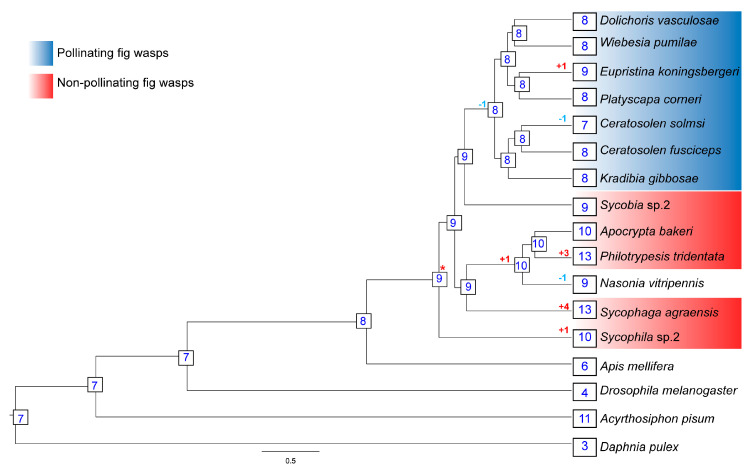
The expansion and contraction of CSP gene family. Numbers on each node are the estimated ancestral copy numbers. The red asterisk represents the number of CSP genes of the most recent common ancestor of the Chalcidoidea.

We used CAFE analyses to estimate the expansion and contraction of CSP gene family in the 11 fig wasps (Figure 5). The lambda value, which represents the probability of gene gain and loss per unit of time during species evolution, was 1.9 × 10^−3^. It was estimated that the most recent common ancestor of the Chalcidoidea had approximately nine CSP genes, and several examples of gene gain and loss were present in specific lineages. For example, there was a net loss of one CSP gene during the evolution of pollinating fig wasps from their common ancestor with *Sycobia* sp.2; the CSP genes in the pollinating fig wasps are a kind of reduced situation. In addition, there was a net gain of three CSP genes during the evolution of *P. tridentata* from its common ancestor with *A. bakeri*; there was a net gain of four CSP genes during the evolution of *S. agraensis* from its common ancestor with *A. bakeri*, *P. tridentata* and *N. vitripennis*.

### 3.7. Codon Adaptation Index Analysis of CSPs to Predict their Expression Indirectly

**Figure 6:**
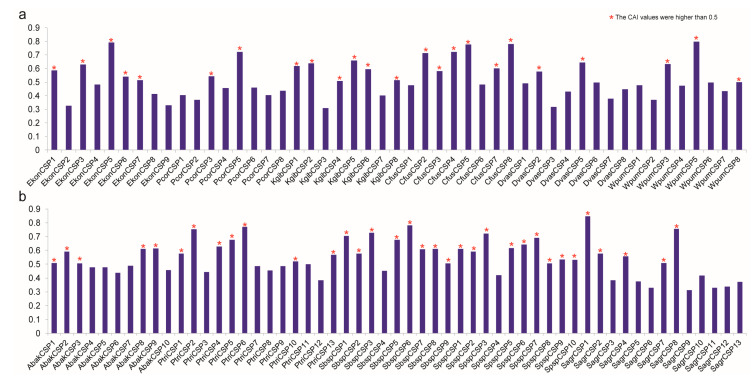
The codon adaptation index (CAI) values of 104 CSP genes in the 11 fig wasps. The red asterisks represent the CSP genes whose CAI values are higher than 0.5. (**a**) The CAI values of CSP genes in six pollinating fig wasps. (**b**) The CAI values of CSP genes in five non-pollinating fig wasps.

CAI is often used to measure gene expression levels; it is a quantitative value that indicates how frequently a favored codon is used amongst highly expressed genes, referring to the coherence of coding region synonymous codons with optimal codon usage frequencies [37,39,40,41]. The working principle of the CAI analysis is to use the sequence of a highly expressed gene as a reference (reference set) to evaluate the degree of codon usage frequency between the target gene and the reference sequence. CAI values range between 0.0 and 1.0, and that the higher the CAI, the stronger the codon use preference and the higher the expression level [42]. A higher CAI value that is higher than 0.5 indicates indirectly that the gene was well expressed, and that a CAI value that is lower than 0.3 is an indicator of low expression indicator [43]. The CAI value is sequence-length independent, depending only on the amino acid frequency [44]. We calculated the CAI values of 104 CSP genes (Figure 6 and Supplementary Materials Figure S2), ranging from 0.310 (KgibCSP3) to 0.847 (SagrCSP1). Among the 104 CSP genes, there was no low-expression gene, since all the CAI values were higher than 0.03, indirectly suggesting that all CSP genes of the 11 fig wasps were highly conserved and might play conservative functions. The CAI values of 58 CSP genes were higher than 0.5, which indirectly indicated that these 58 CSP genes were well expressed. Among these 58 CSP genes, 24 belonged to six pollinating fig wasps and 34 belonged to five non-pollinating fig wasps. The 104 CSP genes were clustered by groups in Supplementary Materials Figure S2.

## 4. Discussion

In this study, we manually annotated 104 CSP genes in the genomes of the 11 fig wasps, all possessing conserved OS-D domains and exhibiting the typical characteristics of insect CSPs, such as the four conserved cysteines and C-pattern (C1-X_6/8_-C2-X_18-19_-C3-X_2_-C4). We comprehensively analyzed these CSP genes from the aspects of gene characteristics, conserved cysteine patterns and motifs orders, phylogeny, distributions on the genome, gene tandem duplications, expansion and contraction, and CAI values. The conclusions drawn mainly include the following two aspects.

### 4.1. The CSP Gene Family is Conserved across the Genomes of Fig Wasps

The 104 CSP genes of the 11 fig wasps are classified into seven groups, based on phylogenetic results (Figure 1). Each of the 11 fig wasps has one CSP gene in five groups (CSPI-CSPV), suggesting that these groups of genes are highly conserved and may play conserved functions in fig wasps. The homologous gene of CSPV group in the *D. melanogaster*, the DmelCSP3, is thought to be involved in the repair and formation of *D. melanogaster* tissues, which may be a target related to embryo and tissue development [45]. Considering that the DmelCSP2 plays a general role in tissue remodeling after injury or during development and is highly expressed during metamorphosis or in response to virus and bacteria [45], we hypothesize that the 11 CSP genes in the CSPIV group closely related to the DmelCSP2 and DmelCSP1 may have similar functions.

The conserved motifs in CSP are an important element of the functional domain. The highly conserved cysteines seem to be the key structure of CSPs, and the motifs patterns can finely tune the function of CSPs, resulting in subtle differences in the binding of different odor molecules [38]. The CSP proteins encoded by the gene members in CSPIII, CSPV and CSPII groups of closely phylogenetic relationships have the same motif orders (8-2-1-3-4) and number of exon (two exons); the CSP proteins encoded by the gene members in the closely related CSPVI and CSPVII groups also have similar motif patterns and number of exon (Figure 3). This pattern indicates that the genes in the closely related groups are highly conserved and may serve similar functions.

CAFE analyses found that the overall numbers of members of CSP gene family are conserved across the chalcidoidea genomes (Figure 5). The number of CSP genes of the most recent common ancestor of the chalcidoidea is very close to the number of CSP genes in the 11 fig wasps genomes. Results of CAI analyses show that the CAI values of CSP genes in CSPIII and CSPV groups are all higher than 0.5 (Figure 6 and Supplementary Materials Figure S2), so these genes can be indirectly considered to be highly expressed; combined with the above analyses, and coupled with the close phylogenetic relationships of CSPIII and CSPV groups, as well as the same motif orders and the numbers of exons, all of these results indicate that these CSP genes are conservative and may have conservative functions.

### 4.2. The CSP Gene Family Is Streamlined in the Pollinating Fig Wasps

Compared to the non-pollinating fig wasps, the pollinating fig wasps have fewer CSP genes. As shown in Figure 4, in the five non-pollinating fig wasps, the number of tandem array varies from two to five, and the number of genes in each tandem array varies from two to five. However, in the six pollinating fig wasps, the numbers of tandem array and genes in each tandem array decrease significantly; the tandem duplication of the CSP gene in the pollinating fig wasps is not obvious. Known mechanisms of gene duplication include tandem duplication, retroposition and segmental (or genome) duplication [46]. Tandem duplications are characterized by multiple members of a family occurring in the same intergenic region or adjacent intergenic regions [47]. Tandem duplication has the advantage of being fast and easy in generating a large number of genes, and it is the most effective mechanism to produce and maintain gene copies [48]. Tandem duplication plays an important role in the origin, generation, maintenance and expansion of gene families [49,50] and has become a common genetic mechanism for organisms to adapt to environmental challenges [51]. Some CSP genes are possibly generated by tandem duplication, and tandem duplication may especially contribute to the increase in the number of CSP genes in the two non-pollinating fig wasps of the *P. tridentata* and *S. agraensis*, which can be further verified from the results of CAFE analyses (Figure 5). For example, the net gain of CSP genes in *P. tridentata* and *S. agraensis* during the evolutionary processes leads to higher numbers of CSP genes in both species compared to other fig wasps. The expansion of the CSP genes in *P. tridentata* and *S. agraensis* may be a species-specific phenomenon, which may link to specific characteristics of both species, especially male polymorphism in the non-pollinating fig wasp of the *P. tridentata*. Further prediction of the approximate gene expression by using the CAI values shows that the number of CSP genes highly expressed in pollinating fig wasps is less than that in non-pollinating fig wasps (24 vs. 34) (Figure 6 and Supplementary Materials Figure S2); this further indicates that CSP genes are in a streamlined state in pollinating fig wasps, since the number of highly expressed CSP genes is smaller when compared to that of non-pollinating fig wasps.

The colonization history of the pollinating fig wasps in the closed and dark syconia is supposed to be longer than most non-pollinating fig wasps [52]. In the long co-evolutionary history, fig syconia provide relatively simple and stable environments for fig wasps, where the pollinating fig wasps live longer in their lives than the non-pollinating fig wasps. Therefore, compared with the non-pollinating fig wasps, or the common ancestor of pollinating fig wasps, pollinating fig wasps may not need too many CSP genes to recognize odorant molecules inside and outside the fig syconia, so the CSP gene family in the pollinating fig wasps is adaptive to be streamlined.

Some insect species, such as *Solenopsis invicta*, *Culex quinquefasciatus*, *Aedes albopictus*, *Nilaparvata lugens*, *Tribolium castaneum*, *Plutella xylostella*, *Bombyx mori* and *Locusta migratoria*, have CSPs gene numbers of 21, 27, 83, 17, 20, 32, 20 and 70, respectively [53,54,55,56,57,58,59] (Table 1); they live in open environments, and their habitats are more complicated than that of the fig syconia. However, the insect species that specifically parasitizes human bodies, *Pediculus humanus*, has only seven CSP genes [60], similar to the pollinating fig wasps in our study; their habitats are relatively concealed and simple. Therefore, we speculate that those insects whose habitats are relatively concealed and simple need fewer CSPs than those who live in open habitats, so the CSP gene family may be in a streamlined state in a simple living environment; that is, the number of CSP genes depends on the complexity of the interaction with the environment [61,62].

## 5. Conclusions

The first comprehensive genome-wide analysis of the CSP gene family in the 11 fig wasp species was carried out in this study. Our results show that the CSP gene family is conserved across the genomes of fig wasps. The reason why pollinating fig wasps have fewer CSP genes than non-pollinating fig wasps may be due to their longer history of adaptation to the fig syconia. We further speculate that the number of CSP genes may be closely related to the complexity of the species’ living environment. These results will provide a good reference for the identification and analysis of CSP genes in other insect species.

## Figures and Tables

**Table 1:** The number of chemosensory proteins (CSP) genes in different insect species.

Species	CSP Gene Number
*Eupristina koningsbergeri*	9
*Platyscapa corneri*	8
*Kradibia gibbosae*	8
*Ceratosolen fusciceps*	8
*Dolichoris vasculosae*	8
*Wiebesia pumilae*	8
*Apocrypta bakeri*	10
*Philotrypesis tridentata*	13
*Sycobia* sp.2	9
*Sycophila* sp.2	10
*Sycophaga agraensis*	13
*Ceratosolen solms*	7
*Nasonia vitripennis*	9
*Apis mellifera*	6
*Drosophila melanogaster*	4
*Solenopsis invicta*	21
*Culex quinquefasciatus*	27
*Aedes albopictus*	83
*Nilaparvata lugens*	17
*Tribolium castaneum*	20
*Plutella xylostella*	32
*Bombyx mori*	20
*Locusta migratoria*	70
*Pediculus humanus*	7

## Supplementary Materials

The following are available online at https://www.mdpi.com/2073-4425/11/10/1149/s1. Figure S1: The relative frequency of multiple CSP amino acids sequences corresponding to each position and multiple amino acid sequences alignment analysis of 104 CSP genes. The four conserved cysteine residues were highlighted with yellow shadow. Figure S2: The CAI values of 104 CSP genes clustered by groups in the 11 fig wasps. The abscissa represented the name of the CSP genes. The ordinate represented the CAI values. The seven CSP groups were distinguished by different colors, the same colors as the seven groups in Figure 1. Table S1: Features of CSP genes identified in the 11 fig wasps.
