# Supplementary material for: Genome-Wide Analysis of Chemosensory Protein Genes (CSPs) Family in Fig Wasps (Hymenoptera, Chalcidoidea)
Source: Genes (Basel). 2020 Sep 29;11(10):1149. doi: 10.3390/genes11101149 (PMC7599541; doi:10.3390/genes11101149)
Supplement: Supplementary file 1 [file genes-11-01149-s001.zip › Supplementary Files/Table S1.docx]

**Table S1.** Features of CSP genes identified in 11 fig wasps.

| **Gene name** | **Scaffold** | **Start** | **End** | **Direction** | **Exon** | **CDS** | **AA** | **%G+C** | **%G+C(1)** | **%G+C(2)** | **%G+C(3)** | **MW(Da)** | **pI** |
| --- | --- | --- | --- | --- | --- | --- | --- | --- | --- | --- | --- | --- | --- |
| EkonCSP1 | scf7180000020258 | 3739191 | 3741367 | - | 2 | 315 | 104 | 48.6 | 42.9 | 35.2 | 67.6 | 11677.9 | 9.4 |
| EkonCSP2 | scf7180000023666 | 2035181 | 2041563 | - | 3 | 390 | 129 | 34.9 | 41.5 | 36.9 | 26.2 | 15146.4 | 8.2 |
| EkonCSP3 | scf7180000020258 | 3298880 | 3300424 | + | 2 | 411 | 136 | 53.3 | 53.3 | 35.8 | 70.8 | 15293.9 | 9.3 |
| EkonCSP4 | scf7180000023666 | 1988306 | 1994014 | - | 3 | 498 | 165 | 48.4 | 50.6 | 40.4 | 54.2 | 19587.5 | 9.2 |
| EkonCSP5 | scf7180000020258 | 3306521 | 3309801 | + | 2 | 390 | 129 | 60.0 | 56.9 | 34.6 | 88.5 | 14672.5 | 4.7 |
| EkonCSP6 | scf7180000020258 | 3726610 | 3729309 | - | 2 | 381 | 126 | 49.3 | 48.8 | 37.0 | 62.2 | 14311.7 | 9.0 |
| EkonCSP7 | scf7180000023666 | 2030536 | 2032916 | + | 2 | 363 | 120 | 43.5 | 45.5 | 28.1 | 57.0 | 14167.4 | 6.8 |
| EkonCSP8 | scf7180000023666 | 2025031 | 2027119 | - | 3 | 504 | 167 | 40.5 | 40.5 | 35.7 | 45.2 | 19626.7 | 5.9 |
| EkonCSP9 | scf7180000020277 | 2246375 | 2248549 | - | 2 | 357 | 118 | 32.8 | 42.0 | 26.1 | 30.3 | 13989.4 | 7.6 |
| PcorCSP1 | scaffold3 | 1739390 | 1743512 | + | 2 | 315 | 104 | 43.2 | 50.5 | 35.2 | 43.8 | 11694.9 | 9.5 |
| PcorCSP2 | scaffold36 | 1838864 | 1842483 | - | 3 | 390 | 129 | 37.9 | 43.1 | 36.9 | 33.8 | 14959.4 | 7.5 |
| PcorCSP3 | scaffold18 | 3032605 | 3034066 | + | 2 | 384 | 127 | 46.9 | 50.0 | 29.7 | 60.9 | 14321.9 | 9.0 |
| PcorCSP4 | scaffold36 | 1791373 | 1797257 | **-** | 3 | 501 | 166 | 48.3 | 52.1 | 40.1 | 52.7 | 19481.4 | 9.3 |
| PcorCSP5 | scaffold18 | 3039567 | 3042360 | + | 2 | 390 | 129 | 59.0 | 56.9 | 40.0 | 80.0 | 14408.2 | 4.9 |
| PcorCSP6 | scaffold3 | 1752555 | 1754928 | + | 2 | 375 | 124 | 44.3 | 50.4 | 36.0 | 46.4 | 14040.4 | 8.9 |
| PcorCSP7 | scaffold36 | 1829941 | 1837070 | + | 2 | 369 | 122 | 40.4 | 45.5 | 34.1 | 41.5 | 14164.6 | 8.2 |
| PcorCSP8 | scaffold36 | 1829377 | 1830169 | - | 2 | 390 | 129 | 42.8 | 50.8 | 33.1 | 44.6 | 14877.2 | 5.4 |
| KgibCSP1 | scafffold1 | 29682557 | 29693331 | - | 2 | 600 | 199 | 59.0 | 54.5 | 48.0 | 74.5 | 22414.9 | 10.3 |
| KgibCSP2 | scafffold1 | 13754216 | 13756072 | + | 2 | 489 | 162 | 53.6 | 47.9 | 41.1 | 71.8 | 18106.0 | 9.6 |
| KgibCSP3 | scafffold1 | 10116831 | 10117471 | - | 2 | 390 | 129 | 33.1 | 40.8 | 36.9 | 21.5 | 15001.4 | 8.1 |
| KgibCSP4 | scafffold1 | 10082839 | 10086998 | - | 3 | 495 | 164 | 50.5 | 53.3 | 39.4 | 58.8 | 19025.9 | 9.2 |
| KgibCSP5 | scafffold1 | 13760998 | 13765431 | + | 2 | 693 | 230 | 68.7 | 67.5 | 57.6 | 81.0 | 24906.9 | 9.6 |
| KgibCSP6 | scafffold1 | 29668775 | 29674372 | - | 2 | 378 | 125 | 51.1 | 52.4 | 34.9 | 65.9 | 14257.8 | 9.3 |
| KgibCSP7 | scafffold1 | 10113239 | 10119410 | + | 2 | 360 | 119 | 38.9 | 45.0 | 33.3 | 38.3 | 13937.3 | 8.9 |
| KgibCSP8 | scafffold1 | 10110778 | 10111479 | - | 2 | 393 | 130 | 46.3 | 48.1 | 35.1 | 55.7 | 14758.0 | 5.9 |
| CfusCSP1 | scaffold4 | 9835292 | 9839214 | - | 2 | 372 | 123 | 46.2 | 46.0 | 35.5 | 57.3 | 14165.7 | 9.8 |
| CfusCSP2 | scaffold10 | 28136 | 29718 | + | 2 | 387 | 128 | 55.6 | 48.1 | 38.0 | 80.6 | 14297.6 | 9.0 |
| CfusCSP3 | scaffold22 | 3287031 | 3288357 | + | 2 | 378 | 125 | 54.8 | 50.0 | 44.4 | 69.8 | 14162.4 | 8.2 |
| CfusCSP4 | scaffold22 | 3313520 | 3316690 | + | 3 | 531 | 176 | 59.7 | 53.7 | 44.1 | 81.4 | 20645.4 | 7.6 |
| CfusCSP5 | scaffold10 | 36519 | 41034 | + | 2 | 489 | 162 | 63.6 | 63.2 | 41.1 | 86.5 | 17863.7 | 4.5 |
| CfusCSP6 | scaffold4 | 9807887 | 9810707 | - | 2 | 372 | 123 | 44.6 | 47.6 | 34.7 | 51.6 | 14108.5 | 9.1 |
| CfusCSP7 | scaffold22 | 3289776 | 3291864 | - | 2 | 714 | 237 | 57.7 | 56.3 | 46.2 | 70.6 | 27245.4 | 9.7 |
| CfusCSP8 | scaffold22 | 3294696 | 3295229 | + | 2 | 399 | 132 | 63.7 | 59.4 | 41.4 | 90.2 | 14906.2 | 8.3 |
| DvasCSP1 | scafffold1 | 19754209 | 19756275 | + | 2 | 309 | 102 | 44.7 | 41.7 | 34.0 | 58.3 | 11731.8 | 9.2 |
| DvasCSP2 | scafffold1 | 7358192 | 7359652 | - | 2 | 384 | 127 | 48.7 | 48.4 | 34.4 | 63.3 | 14216.6 | 9.2 |
| DvasCSP3 | scafffold1 | 1795925 | 1802045 | - | 3 | 525 | 174 | 34.9 | 40.0 | 36.6 | 28.0 | 20285.4 | 7.5 |
| DvasCSP4 | scafffold1 | 1751646 | 1757855 | - | 3 | 498 | 165 | 44.8 | 50.6 | 38.0 | 45.8 | 19495.5 | 9.0 |
| DvasCSP5 | scafffold1 | 7349564 | 7351798 | - | 2 | 456 | 151 | 59.9 | 63.8 | 40.1 | 75.7 | 16777.9 | 5.0 |
| DvasCSP6 | scafffold1 | 19764809 | 19768007 | + | 2 | 378 | 125 | 48.1 | 54.0 | 34.1 | 56.3 | 14289.7 | 9.0 |
| DvasCSP7 | scafffold1 | 1791142 | 1793797 | + | 2 | 366 | 121 | 38.8 | 44.3 | 32.8 | 39.3 | 14078.3 | 7.6 |
| DvasCSP8 | scafffold1 | 1787717 | 1788498 | - | 2 | 414 | 137 | 42.3 | 41.3 | 35.5 | 50.0 | 15809.5 | 8.8 |
| WpumCSP1 | scaffold44 | 539664 | 542329 | - | 2 | 315 | 104 | 45.7 | 46.7 | 37.1 | 53.3 | 11620.8 | 9.6 |
| WpumCSP2 | scaffold14 | 1746936 | 1751189 | - | 3 | 384 | 127 | 35.7 | 39.1 | 33.6 | 34.4 | 14693.3 | 6.2 |
| WpumCSP3 | scaffold40 | 1725691 | 1727086 | + | 2 | 387 | 128 | 52.2 | 48.8 | 36.4 | 71.3 | 14230.7 | 9.1 |
| WpumCSP4 | scaffold14 | 1704083 | 1709676 | - | 3 | 498 | 165 | 48.2 | 51.2 | 39.8 | 53.6 | 19456.3 | 9.0 |
| WpumCSP5 | scaffold40 | 1733663 | 1735701 | + | 2 | 384 | 127 | 64.8 | 60.2 | 43.8 | 90.6 | 13991.8 | 5.0 |
| WpumCSP6 | scaffold44 | 526212 | 529730 | - | 2 | 375 | 124 | 47.5 | 52.0 | 36.8 | 53.6 | 13982.3 | 8.9 |
| WpumCSP7 | scaffold14 | 1743177 | 1744750 | + | 2 | 369 | 122 | 42.0 | 43.9 | 35.8 | 46.3 | 13998.1 | 8.8 |
| WpumCSP8 | scaffold14 | 1738410 | 1740068 | - | 2 | 495 | 164 | 47.3 | 47.9 | 37.0 | 57.0 | 18724.4 | 5.7 |
| AbakCSP1 | scaffold14 | 2088837 | 2091461 | - | 2 | 300 | 99 | 48.3 | 51.0 | 38.0 | 56.0 | 11038.1 | 9.3 |
| AbakCSP2 | scaffold9 | 962212 | 962865 | + | 2 | 387 | 128 | 48.1 | 48.1 | 33.3 | 62.8 | 14699.1 | 8.9 |
| AbakCSP3 | scaffold12 | 913415 | 919793 | - | 2 | 360 | 119 | 42.5 | 44.2 | 31.7 | 51.7 | 13803.1 | 8.3 |

**Table S1.** *Cont.*

| **Gene name** | **Scaffold** | **Start** | **End** | **Direction** | **Exon** | **CDS** | **AA** | **%G+C** | **%G+C(1)** | **%G+C(2)** | **%G+C(3)** | **MW(Da)** | **pI** |
| --- | --- | --- | --- | --- | --- | --- | --- | --- | --- | --- | --- | --- | --- |
| AbakCSP4 | scaffold12 | 911642 | 912622 | + | 2 | 390 | 129 | 46.4 | 44.6 | 43.1 | 51.5 | 14565.9 | 8.8 |
| AbakCSP5 | scaffold12 | 918827 | 919298 | + | 2 | 381 | 126 | 48.6 | 51.2 | 40.9 | 53.5 | 14090.2 | 5.4 |
| AbakCSP6 | scaffold12 | 920759 | 924044 | - | 2 | 405 | 134 | 45.7 | 52.6 | 41.5 | 43.0 | 15134.4 | 7.6 |
| AbakCSP7 | scaffold12 | 957455 | 962921 | + | 3 | 552 | 183 | 49.1 | 49.5 | 44.0 | 53.8 | 21226.5 | 9.8 |
| AbakCSP8 | scaffold14 | 2083435 | 2085457 | - | 2 | 372 | 123 | 50.3 | 48.4 | 36.3 | 66.1 | 14060.4 | 9.0 |
| AbakCSP9 | scaffold9 | 967212 | 968417 | + | 2 | 438 | 145 | 53.7 | 55.5 | 37.7 | 67.8 | 16322.3 | 4.8 |
| AbakCSP10 | scaffold12 | 962739 | 965401 | - | 2 | 414 | 137 | 44.4 | 52.2 | 31.2 | 50.0 | 15742.5 | 8.1 |
| PtriCSP1 | scaffold16 | 1632426 | 1634744 | + | 2 | 321 | 106 | 55.1 | 58.9 | 39.3 | 67.3 | 11802.9 | 9.2 |
| PtriCSP2 | scaffold16 | 1639333 | 1641473 | + | 2 | 375 | 124 | 60.5 | 55.2 | 40.8 | 85.6 | 13930.3 | 9.0 |
| PtriCSP3 | scaffold16 | 2187028 | 2188388 | - | 3 | 402 | 133 | 42.3 | 43.3 | 37.3 | 46.3 | 15357.7 | 6.5 |
| PtriCSP4 | scaffold5 | 4596357 | 4597073 | + | 2 | 390 | 129 | 51.8 | 53.8 | 34.6 | 66.9 | 14702.0 | 8.5 |
| PtriCSP5 | scaffold16 | 2135866 | 2139558 | - | 3 | 606 | 201 | 58.3 | 50.0 | 45.5 | 79.2 | 23161.4 | 9.7 |
| PtriCSP6 | scaffold5 | 4604247 | 4605577 | + | 2 | 459 | 152 | 64.3 | 64.7 | 41.2 | 86.9 | 16734.4 | 4.6 |
| PtriCSP7 | scaffold16 | 2183916 | 2185187 | + | 2 | 363 | 120 | 43.0 | 44.6 | 33.1 | 51.2 | 13824.1 | 8.6 |
| PtriCSP8 | scaffold16 | 2251300 | 2252002 | - | 2 | 366 | 121 | 40.4 | 43.4 | 29.5 | 48.4 | 13965.5 | 6.3 |
| PtriCSP9 | scaffold16 | 2164970 | 2166211 | + | 2 | 528 | 175 | 47.3 | 51.7 | 37.5 | 52.8 | 20193.5 | 8.1 |
| PtriCSP10 | scaffold16 | 2175149 | 2178655 | + | 2 | 360 | 119 | 44.7 | 43.3 | 35.0 | 55.8 | 13559.8 | 8.2 |
| PtriCSP11 | scaffold16 | 2167318 | 2167814 | - | 2 | 381 | 126 | 47.0 | 48.8 | 36.2 | 55.9 | 14416.7 | 6.6 |
| PtriCSP12 | scaffold35 | 729615 | 730236 | - | 2 | 378 | 125 | 34.9 | 37.3 | 30.2 | 37.3 | 14777.3 | 9.1 |
| PtriCSP13 | scaffold16 | 2134096 | 2135477 | + | 2 | 423 | 140 | 52.7 | 54.6 | 35.5 | 68.1 | 16084.9 | 8.1 |
| SbspCSP1 | scaffold12 | 4229311 | 4231545 | - | 2 | 327 | 108 | 55.7 | 48.6 | 36.7 | 81.7 | 12316.7 | 9.8 |
| SbspCSP2 | scaffold12 | 4200953 | 4203195 | - | 2 | 381 | 126 | 53.0 | 53.5 | 38.6 | 66.9 | 14044.2 | 8.9 |
| SbspCSP3 | scaffold5 | 6643405 | 6645960 | + | 2 | 408 | 135 | 56.6 | 54.4 | 35.3 | 80.1 | 15228.7 | 8.2 |
| SbspCSP4 | scaffold116 | 12683 | 13299 | - | 2 | 483 | 160 | 41.8 | 40.4 | 34.8 | 50.3 | 18983.2 | 8.9 |
| SbspCSP5 | scaffold36 | 140476 | 150620 | + | 3 | 495 | 164 | 57.6 | 52.7 | 43.6 | 76.4 | 19160.1 | 9.3 |
| SbspCSP6 | scaffold5 | 6661427 | 6665909 | + | 2 | 462 | 153 | 71.2 | 70.1 | 49.4 | 94.2 | 16281.0 | 4.4 |
| SbspCSP7 | scaffold36 | 22947 | 26489 | - | 2 | 342 | 113 | 49.7 | 44.7 | 35.1 | 69.3 | 13126.1 | 8.2 |
| SbspCSP8 | scaffold37 | 1352299 | 1353989 | + | 2 | 366 | 121 | 48.1 | 47.5 | 27.9 | 68.9 | 14125.6 | 8.5 |
| SbspCSP9 | scaffold36 | 148755 | 152404 | - | 2 | 393 | 130 | 47.1 | 49.6 | 35.9 | 55.7 | 15025.6 | 9.0 |
| SpspCSP1 | scaffold31 | 33037 | 35438 | + | 2 | 312 | 103 | 54.8 | 55.8 | 36.5 | 72.1 | 11692.0 | 10.2 |
| SpspCSP2 | scaffold31 | 341000 | 345629 | + | 2 | 375 | 124 | 51.7 | 53.6 | 34.4 | 67.2 | 14029.4 | 9.1 |
| SpspCSP3 | scaffold6 | 445909 | 446886 | - | 2 | 387 | 128 | 55.3 | 51.9 | 38.0 | 76.0 | 14521.8 | 8.9 |
| SpspCSP4 | scaffold36 | 2005874 | 2007029 | - | 2 | 474 | 157 | 42.2 | 41.8 | 43.0 | 41.8 | 17387.9 | 7.5 |
| SpspCSP5 | scaffold36 | 1966087 | 1969452 | - | 3 | 510 | 169 | 58.0 | 56.5 | 44.7 | 72.9 | 19643.6 | 9.1 |
| SpspCSP6 | scaffold36 | 2003667 | 2005041 | + | 2 | 351 | 116 | 51.6 | 48.7 | 34.2 | 71.8 | 13288.4 | 8.2 |
| SpspCSP7 | scaffold6 | 440565 | 441524 | - | 2 | 468 | 155 | 61.8 | 61.5 | 42.9 | 80.8 | 17573.9 | 5.4 |
| SpspCSP8 | scaffold36 | 1998797 | 2000005 | + | 4 | 423 | 140 | 47.8 | 53.2 | 35.5 | 54.6 | 16255.7 | 6.3 |
| SpspCSP9 | scaffold36 | 2000651 | 2001669 | - | 2 | 654 | 217 | 53.7 | 51.4 | 46.8 | 62.8 | 24185.3 | 6.2 |
| SpspCSP10 | scaffold36 | 1964072 | 1965715 | + | 2 | 426 | 141 | 51.9 | 61.3 | 35.2 | 59.2 | 15995.6 | 5.8 |
| SagrCSP1 | scaffold14 | 3083368 | 3086211 | + | 2 | 315 | 104 | 63.8 | 58.1 | 38.1 | 95.2 | 11769.9 | 9.8 |
| SagrCSP2 | scaffold14 | 3090828 | 3094627 | + | 2 | 369 | 122 | 52.0 | 52.0 | 39.0 | 65.0 | 13788.1 | 9.1 |
| SagrCSP3 | scaffold123 | 240922 | 242362 | + | 3 | 390 | 129 | 40.5 | 45.4 | 36.2 | 40.0 | 14953.4 | 8.2 |
| SagrCSP4 | scaffold15 | 690736 | 694946 | - | 3 | 504 | 167 | 53.6 | 53.6 | 42.3 | 64.9 | 19771.0 | 9.6 |
| SagrCSP5 | scaffold15 | 735411 | 736947 | + | 2 | 366 | 121 | 35.5 | 38.5 | 30.3 | 37.7 | 14084.5 | 8.6 |
| SagrCSP6 | scaffold15 | 728717 | 732759 | + | 2 | 405 | 134 | 35.8 | 44.4 | 35.6 | 27.4 | 15536.0 | 8.9 |
| SagrCSP7 | scaffold2 | 5915601 | 5916204 | - | 2 | 390 | 129 | 46.7 | 49.2 | 36.2 | 54.6 | 14598.9 | 9.0 |
| SagrCSP8 | scaffold2 | 5903076 | 5905616 | - | 2 | 399 | 132 | 66.2 | 63.2 | 46.6 | 88.7 | 14714.8 | 8.7 |
| SagrCSP9 | scaffold15 | 739621 | 740244 | + | 2 | 345 | 114 | 31.0 | 38.3 | 27.8 | 27.0 | 13305.5 | 5.5 |
| SagrCSP10 | scaffold15 | 732346 | 733586 | - | 2 | 366 | 121 | 42.6 | 49.2 | 36.1 | 42.6 | 13879.9 | 5.7 |
| SagrCSP11 | scaffold10 | 2387059 | 2388053 | - | 2 | 345 | 114 | 32.8 | 40.9 | 27.8 | 29.6 | 13369.6 | 5.3 |
| SagrCSP12 | scaffold10 | 2391898 | 2392893 | - | 2 | 345 | 114 | 33.6 | 42.6 | 27.0 | 31.3 | 13248.6 | 5.2 |
| SagrCSP13 | scaffold15 | 689116 | 691377 | + | 2 | 414 | 137 | 36.5 | 47.8 | 26.8 | 34.8 | 16162.9 | 7.6 |
