# Supplementary figures and images for: Genome-Wide Analysis of Chemosensory Protein Genes (CSPs) Family in Fig Wasps (Hymenoptera, Chalcidoidea)
Source: Genes (Basel). 2020 Sep 29;11(10):1149. doi: 10.3390/genes11101149 (PMC7599541; doi:10.3390/genes11101149)

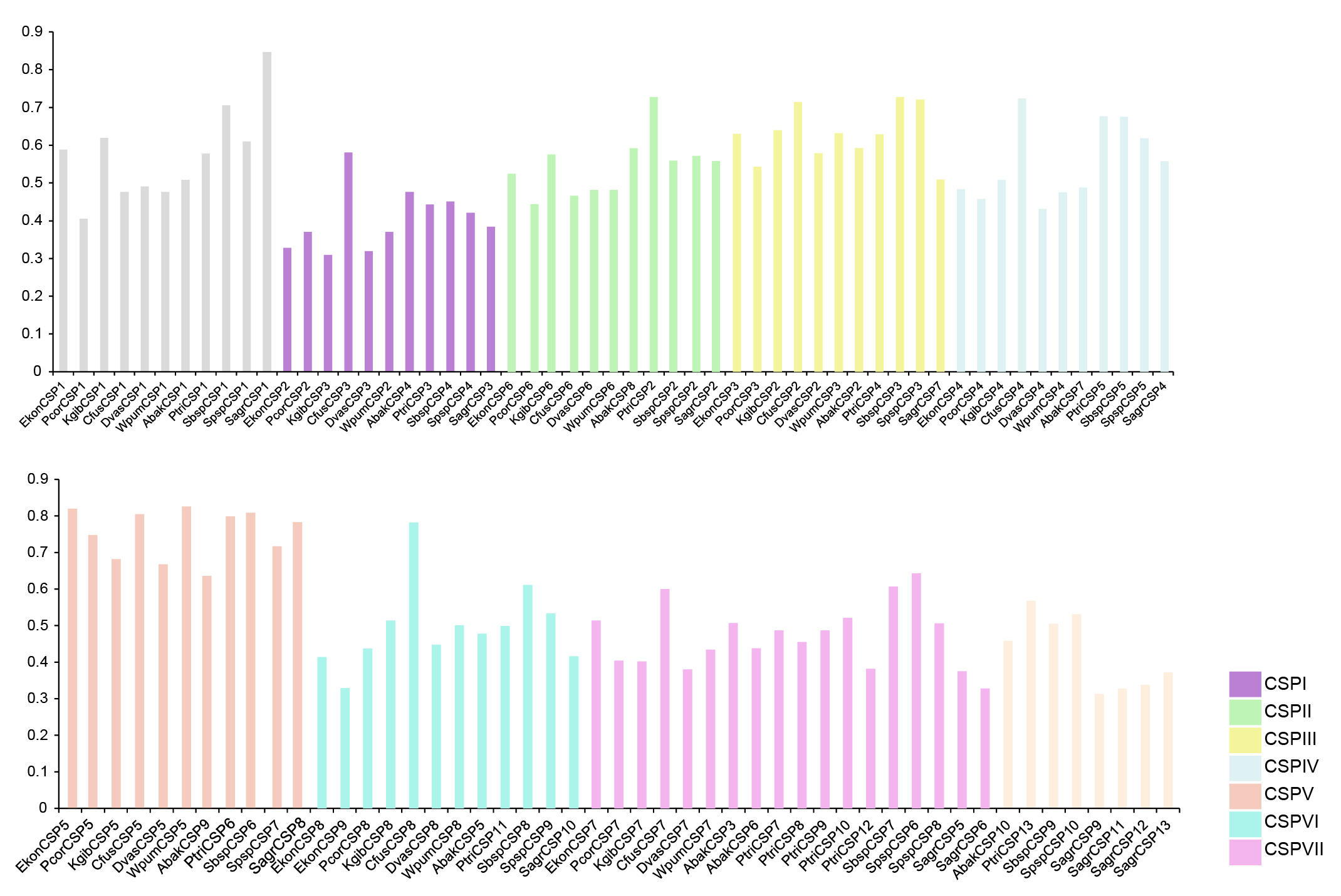

Supplement: Supplementary file 1 [file genes-11-01149-s001.zip › Supplementary Files/Figure S2.tif]
